# 4320 Acral, Head and Neck Melanoma Subtype Classification Performance Using A Convolutional Neural Network (CNN) Trained On a Public Dataset

**DOI:** 10.1017/cts.2020.165

**Published:** 2020-07-29

**Authors:** Payal Shah, Sameer Arya, Lauren Rangel, Yindalon Aphinyanaphongs

**Affiliations:** 1 New York University Langone Health

## Abstract

OBJECTIVES/GOALS: Composition of demographics or image types in publicly available datasets may detract from deep learning (DL) diagnosis performance of underrepresented melanoma subtypes. We evaluate a DL model’s performance on melanoma subtypes (acral; head and neck) that have known association with poor prognosis. METHODS/STUDY POPULATION: We trained a CNN using a single InceptionV3 model for 30 epochs on dermoscopic images of pigmented lesions from the International Skin Imaging Collaboration (ISIC). The ISIC 2018 challenge training set had 10008 total images, with 1113 total nevi, 6705 total melanomas, 97 acral nevi, 10 acral melanomas, 256 head and neck (H&N) nevi, and 164 H&N melanomas. The non-acral test set had 117 melanomas and 200 nevi. The acral test set had 201 melanomas and 161 nevi. The H&N test set had 199 melanomas and 128 nevi. Area under the receiver operating curve (AUC) was calculated. The model was retrained with acral lesion oversampling (10x) and performance on the acral test set was re-evaluated. RESULTS/ANTICIPATED RESULTS: The model performed on the non-acral test with an AUC of 80.5%, on the acral test with an AUC of 76.3%, and on the head and neck test with an AUC of 83.8% After oversampling acral lesions within the training set, the model showed nearly the same performance as without oversampling on acral lesions: AUC of 75.6%. DISCUSSION/SIGNIFICANCE OF IMPACT: Diagnosis of high-risk melanoma subsets (acral; H&N) remains reliable despite underrepresentation during training, increasing validity for broad implementation of DL technology. Datasets for individual subtypes may not be warranted as findings suggest features may be learned from other skin lesions.

